# Schaftoside contributed to anti-inflammatory activity of *Clinacanthus nutans* extract in lipopolysaccharide-induced RAW 264.7 cells

**DOI:** 10.3389/fphar.2025.1584620

**Published:** 2025-06-10

**Authors:** Saruda Thongyim, Pachara Sattayawat, Wittaya Pongamornkul, Siriphorn Jangsutthivorawat, Yingmanee Tragoolpua, Aussara Panya

**Affiliations:** ^1^ Office of Research Administration, Chiang Mai University, Chiang Mai, Thailand; ^2^ National Extracts and Innovative Products for Alternative Healthcare Research Group, Chiang Mai University, Chiang Mai, Thailand; ^3^ Department of Biology, Faculty of Science, Chiang Mai University, Chiang Mai, Thailand; ^4^ Cell Engineering for Cancer Therapy Research Group, Faculty of Science, Chiang Mai University, Chiang Mai, Thailand; ^5^ Queen Sirikit Botanic Garden Organization, Chiang Mai, Thailand

**Keywords:** inflammation, *Clinacanthus nutans*, Herb extract, schaftoside, RAW 264.7 cells

## Abstract

**Introduction:**

*Clinacanthus nutans*: a plant listed in the Thai Herbal Pharmacopoeia, is well recognized for its medicinal properties, particularly its anti-inflammatory and antiviral activities. Among its known bioactive constituents, schaftoside has been reported to exhibit anti-inflammatory effects in various disease models. However, comparative studies between pure schaftoside and *C. nutans* crude extracts, as well as comprehensive investigations into the underlying mechanisms of action, remain limited. Moreover, the relationship between the quantity and diversity of bioactive compounds and their corresponding anti-inflammatory activity which could serve as potential quality biomarkers has not been fully elucidated.

**Methods:**

In this study, we investigated the anti-inflammatory effects of schaftoside and evaluated its content in *C. nutans* ethanolic extracts collected from ten geographically distinct regions of Thailand. First, the anti-inflammatory activity of schaftoside was assessed in LPS-induced RAW 264.7 macrophage cells. Subsequently, ten *C. nutans* ethanolic extracts were tested for their anti-inflammatory activity in the same cell model. To further explore the potential contribution of schaftoside and other bioactive compounds to anti-inflammatory activity, molecular docking analysis was performed.

**Results and Discussion:**

At a concentration of 40 μM, schaftoside significantly downregulated the expression of key inflammation-related genes, including *iNOS, COX2, PGE2, PGE4, TNF-α, and IL6*. All extracts demonstrated a consistent trend of reducing iNOS protein expression, which was accompanied by a corresponding decrease in nitric oxide (NO) production, indicating their potential anti-inflammatory properties. However, no significant correlation was observed between schaftoside content and the magnitude of anti-inflammatory activity, suggesting that schaftoside may not be the sole active compound responsible for the observed effects. The results of molecular docking analysis revealed that, in addition to schaftoside, other flavonoids such as isoorientin and isovitexin also exhibited binding affinity toward the iNOS protein, indicating that these compounds may contribute to the overall anti-inflammatory activity of *C. nutans* extracts.

## 1 Introduction

*Clinacanthus nutans (Burm. f.)* Lindau, or Phaya Yo, is a plant listed in the Thai Pharmacopoeia and is widely used in folk medicine for treating health conditions, particularly skin related such as snake or insect bites (Alam et al., 2016). Several bioactive compounds were reported from *C. nutans* for several bioactivities including antiviral (Jantakee et al., 2024; Lin et al., 2023), antioxidant (Ismail et al., 2020; Rahman et al., 2019), antidiabetic (Pechroj et al., 2024), anticancer (Mutazah et al., 2020), antimicrobial (Chiangchin et al., 2023) and anti-inflammatory (Thongyim et al., 2024) properties. Ethanolic extracts of *C. nutans* that have been shown for anti-inflammations and different bioactive compounds including vitexin, isoorientin, orientin, isovitexin and schaftoside were mentioned (Ong et al., 2022).

Genetic variation and environmental factors can influence the phytochemical profile and content of plant extracts, thereby affecting their biological activity. Although the biological activity of *C. nutans* has been well documented, only limited studies have explored the correlation between its genetic background, phytochemical profile, and biological activity. Recently, our group investigated the genetic variation of *C. nutans* from ten different regions of Thailand using sequence-related amplified polymorphism (SRAP) markers. The results, analyzed using UPGMA cluster analysis, showed that all samples were closely related (Chiangchin et al., 2023). These findings highlight the greater influence of environmental factors reflected in the phytochemical profile on the biological activity of *C. nutans*. Furthermore, understanding the relationship between the quantity of candidate bioactive compounds and their corresponding anti-inflammatory activity could help identify potential quality biomarkers. Such biomarkers could improve the therapeutic and commercial quality of *C. nutans*, while also supporting sustainable conservation efforts.

Among these, schaftoside was the focus due to its report as a major flavone present in *C. nutans* ethanolic extracts (Chelyn et al., 2014). Apart from *C. nutans* (Ong et al., 2022), schaftoside (6-C-b-glucopyranosyl-8-C-a-arabinopyranosylapigenin) is a bioactive constitute in many medicinal herbs including *Eleusine indica* or Indian Goosegrass (De Melo et al., 2005) and *Grona styracifolia* or Guang Jing Qian Cao (Zeng et al., 2024) and *Desmodium styracifolium* or Coin-leaf desmodium (Liu et al., 2020). In one study, inflammatory responses via interleukin 6 (IL-6), interleukin 1 beta (IL-1β), and nuclear factor kappa-light-chain-enhancer of activated B cells (NF-κB) were downregulated upon schaftoside treatment in zebrafish (Dang et al., 2021). Moreover, De Melo and colleagues reported that treatment of 400 μg/kg schaftoside extracted from *Eleusine indica* inhibited 62% of lung neutrophil influx in mice exposed to aerosols of lipopolysaccharide (LPS) from gram-negative bacteria emphasizing schaftoside possesses anti-inflammatory properties (De Melo et al., 2005). Inflammation-related signaling pathways are well documented, with the NF-κB pathway recognized as a central regulator (Liu et al., 2017). Schaftoside has been shown to inhibit NF-κB signaling (Liu et al., 2020); however, the detailed mechanisms underlying its anti-inflammatory effects, as well as the relationship between schaftoside content in *C. nutans* extracts and their anti-inflammatory activity, remain unclear.

**FIGURE 1:**
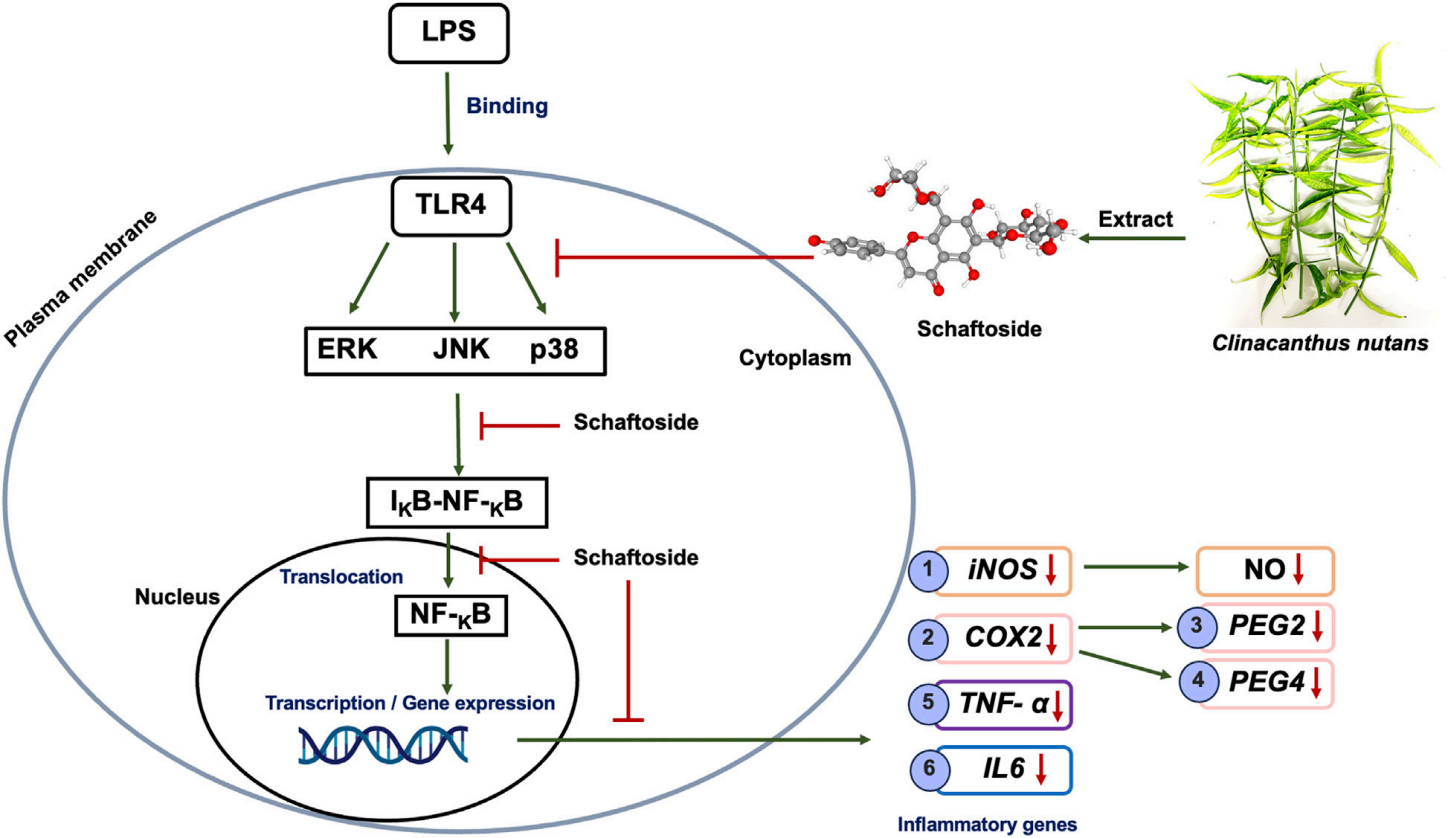
Schematic representation of the proposed mechanism of schaftoside anti-inflammation effects through the NF-κB signaling pathway in LPS-induced inflammation on RAW 264.7 cell lines.

Based on this, we hypothesized that schaftoside is a key bioactive compound in *C. nutans* extracts contributing to anti-inflammatory activity through inhibition of the NF-κB signaling pathway (Figure 1). We further proposed that variations in schaftoside content would positively correlate with the degree of anti-inflammatory activity in extracts collected from different regions of Thailand. To test this hypothesis, we examined the anti-inflammatory mechanisms of schaftoside at both mRNA and protein expression levels in LPS-stimulated RAW 264.7 cells. The RAW 264.7 murine macrophage cell line is extensively utilized as an *in vitro* model for investigating inflammation, owing to its macrophage-like characteristics, including robust production of pro-inflammatory mediators such as nitric oxide and cytokines upon LPS stimulation. Its ease of cultivation, well-characterized phenotype, and consistent inflammatory responses make it a reliable and reproducible system for elucidating inflammatory pathways and evaluating potential anti-inflammatory agents (Gu et al., 2025; González-Montoya et al., 2022; Kim et al., 2013; Zhang et al., 2018; Cifuentes et al., 2018).

In this study, *C. nutans* extracts from ten distinct locations across Thailand were evaluated for their anti-inflammatory activity to assess the correlation between schaftoside content and bioactivity, as well as to compare variation in activity among the samples. Further analysis was conducted using molecular docking to explore other potential bioactive compounds present in *C. nutans* ethanolic extracts. This approach identified additional candidates that may contribute to the plant’s anti-inflammatory properties, providing valuable insights for future research and broadening the scope for therapeutic development.

## 2 Materials and methods

### 2.1 *Clinacanthus nutans* (Phaya Yo) extraction

Ten samples of *Clinacanthus nutans* were collected from different locations in Thailand following the protocol which previously described (Chiangchin et al., 2023). The plants were deposited at the Queen Sirikit Botanic Garden Herbarium (QSBG herbarium), Chiang Mai, Thailand (Supplementary Table S1). Briefly, the fresh leaves of *C. nutans* were cleaned with water, dried at 60°C in a hot air oven for 48–72 h and blended into powder with a blender before resuspending in 95% ethanol (1:20 (w/v)). After that, all samples were shaken at 160 rpm/min at room temperature for 72 h and the 95% ethanol was changed every 24 h. The samples were filtered by Whatman No. One and the liquid fraction was evaporated in a rotary evaporator at 45°C. The 95% ethanol crude extracts were then dried in a chemical fume hood until completely dried and stored at 20°C until use.

### 2.2 Cell culture and reagents

A macrophage cell line of murine (RAW 264.7 cells), was purchased from ATCC with the accession number ATCC-TIB-71 and were cultured in Dulbecco’s Modified Eagle Medium (DMEM high glucose, Gibco, Invitrogen Life Technologies, MA, United States) supplemented with 10% (v/v) fetal bovine serum (FBS), 1% (v/v) L-glutamine, and 1% (v/v) antibiotic (penicillin G, streptomycin) and incubated at 37°C in a humidified 5% CO_2_ incubator. The cells were sub-cultured twice a week.

### 2.3 Cell viability assay

To determine the % cell viability of RAW 264.7 cells after *C. nutans* treatment, RAW 264.7 cells were plated a day before the experiment at 100,000 cells/well in the 96 well plates. The cells were then treated with *C. nutans* ethanolic extract at concentrations of 0.8–500 μg/mL (5-fold dilution) and schaftoside at concentrations of 6.25–200 μg/mL (2-fold dilution). The LPS was used to induce inflammation by treating at a concentration of 1 × 10^-4^–10 μg/mL (10-fold dilution). After that, the cells incubated at the same condition for 24 and 48 h. The percentage viability of the cells when treated with *C. nutans*, schaftoside, and LPS was determined by using prestoBLUE™ cell viability reagent (Invitrogen, MA, United States). The absorbance at OD 570 and OD 595 was measured using a microplate reader (DYNEX Technologies, Chantilly, VA, United States). The % cell viability was calculated and compared to that of the control (non-treated cells), as formula below.

$$\%\text{ Cell viability}=\frac{\left(\text{OD}570-\text{OD}595\right)\;\text{treated}\;\text{cells}}{\left(\text{OD}570-\text{OD}595\right)\;\text{non}-\text{treated}\;\text{cells}}x\;100\%$$

The cytotoxicity of *E. coli* LPS (L4391-1 MG, *E. coli* 0111: B4, Sigma- Aldrich, St. Louis, MO, United States) was also investigated by which the LPS at concentrations of 1 × 10^-4^–10 μg/mL were used to treat the cells and the % cell viability was assessed using prestoBLUE™ cell viability reagent as mentioned. This screening assay was to determine the effect of LPS that could induce cell deaths and inflammation for use in further experiments. The selected final concentration of LPS was 1 μg/mL.

### 2.4 Immunoblotting

Firstly, RAW 264.7 cells were plated into 24-well plates at a density of 500,000 cells/well a day before the experiment. The total protein was prepared after LPS (1 μg/mL) treatment in combination with *C. nutans* (300 μg/mL) in each sample or schaftoside (10–40 μM) for 24 h using 1XRSB. The proteins were separated via 12% SDS-PAGE and transferred onto a nitrocellulose membrane. The immunoblotting detection using the antibodies specific to iNOS and GAPDH (at the dilution 1:1,000) were purchased from cell signaling (MA, United States). The proteins were detected using a secondary antibody goat anti-rabbit (at the dilution 1:1,000) purchased from ABclonal (MA, United States). The protein bands were visualized using SuperSignal West Pico PLUS Chemiluminescent Substrate purchased by Thermo Fisher Scientific (Waltham, MA, United States). Visualization of target proteins was detected through ImageQuant™ LAS 500 Chemiluminescent Imaging System (GE Healthcare, Chicago, IL, United States). The protein band intensity was analyzed using the ImageJ program. GAPDH proteins were housekeeping proteins to normalize the fold change of protein.

### 2.5 NO production

The RAW 264.7 cells were plated into 24-well plates at a density of 500,000 cells/well a day before the experiment. LPS was used to induce inflammation of the cells at a concentration of 1 μg/mL combined with treatment of *C. nutans* (300 μg/mL) in each sample and schaftoside (10–40 μM) for 24 h. The amount of nitric oxide in the supernatant was determined using a Gress reaction assay. The absorbance was measured using a microplate reader at 540 nm. The NO production level was compared to that non-treatment control (the control was set as 1.0).

### 2.6 Gene expression by real time PCR

To determine the effect of schaftoside on reducing inflammation in RAW 264.7 cells by using real-time PCR. Briefly, the cells were plated in a 12-well plate at a density of 500,000 cells/well and treated with LPS (1 μg/mL) with schaftoside (40 μM) for 24 h. *C. nutans* (300 μg/mL) with LPS (1 μg/mL) was used as a positive control in this experiment. Total RNA was isolated from RAW 264.7 cells using TRIzol^®^ reagent (Invitrogen Life Technologies, MA, United States). The total RNA was converted into cDNA using the Tetro cDNA Synthesis Kit (Bioline United States Inc., United States). The cDNA was used for real time PCR using Luna^®^ Universal qPCR Master Mix (New England Biolabs, MA, United States) for analysis of the inflammatory genes including *IL6*, *TNF-alpha*, *iNOS*, *COX2*, *PGE2*, and *PGE4*. The realtime PCR was conducted in QIAquant real-time PCR cyclers (Qiagen, Venlo, Netherlands) using specific primes (Supplementary Table 1). The level of mRNA of inflammatory genes was calculated with the 2^−ΔΔCT^ method. GADPH was used as the housekeeping gene for the normalization of gene expression and compared relative to that of LPS (non-treated with schaftoside or *C. nutans* extracts), set as 100.

### 2.7 HPLC analysis

To investigate the schaftoside in *C. nutans* leaf extracts using high-performance liquid chromatography (HPLC). The sample of *C. nutans* extracts was dissolved in methanol (HPLC grade) at a concentration of 10 mg/mL and was filtrated through a 0.45 μm of microfilter. After that, all samples were run through an HPLC equipped with Agilent Zorbax Eclipse XDB - C18 column (5 μm, 4.6 × 150 mm, GL Science, Tokyo, Japan). The 10 μL of *C. nutans* extracts solution was injected using an isocratic HPLC system in which solution A: 0.1% formic acid and solution B: acetonitrile (85:15; v/v) were used as the mobile phase, flow rate 1 mL/min for 15 min at 35°C. The UV absorption was monitored at 340 nm. The amount of schaftoside in *C. nutans* extracts was calculated by comparing with the standard curve of schaftoside (Chem Faces Biochemical Co., Ltd., Wuhan, China).

### 2.8 Total phenolic content

The total phenolic content of *C. nutans* leaf extracts were investigated using the Folin-Ciocalteu method. Briefly, *C. nutans* extracts (25 μL in 96-well plates) were mixed with 125 μL of distilled water, followed by 25 μL of 95% ethanol, and 12.5 μL of Folin-Ciocalteu (50% (v/v)). After that, the plate was incubated at room temperature for 5 min (Dark incubation) and 25 μL of sodium carbonate (5% (w/v)) was added. After incubation at room temperature for 1 h in the dark, the absorbance was measured at 725 nm using a microplate reader. The amount of total phenolic compound in *C. nutans* extracts was calculated by comparing with the standard curve of gallic acid (TCI AMERICA, Portland, OR, United States) and was expressed as mg of gallic acid equivalents (GAE)/1 g of *C. nutans* extracts (mg GAE/g extracts).

### 2.9 Molecular docking analysis

Molecular docking analysis was performed using GOLD Protein–ligand docking software (Verdonk et al., 2003). The iNOS protein structure was downloaded from PDB databank with a PDB ID IQW4. The chemical structures of schaftoside, vitexin, isoorientin, orientin and isovitexin were obtained from PubChem databank (https://www.rcsb.org). The docking site was selected to be the same site with ligand A co-crystallized with the protein structure according to a previous study that concluded the interactions of iNOS with inhibitors to be GLU371, TYR367, ASH376 and ARG382 (Minhas and Bansal, 2022). CHEMPLP score was used to determine the binding.

The best docking poses were further analyzed using Discovery Studio Visualizer (BIOVIA, 2017).

### 2.10 Statistical analysis

The bar graphs representing the mean and standard error of the mean (SEM) were plotted from three independent measurements. The statistical analyses were performed using Student’s t-test of GraphPad Prism Software version 10 (GraphPad Software, Inc., La Jolla, CA, United States), where significant differences were indicated as follows: (ns) indicates *p* > 0.05, *p < 0.05, **p < 0.01, ***p < 0.001, ****p < 0.0001.

## 3 Results

### 3.1 Schaftoside reduced inflammation in mRNA and protein expression levels

**FIGURE 2:**
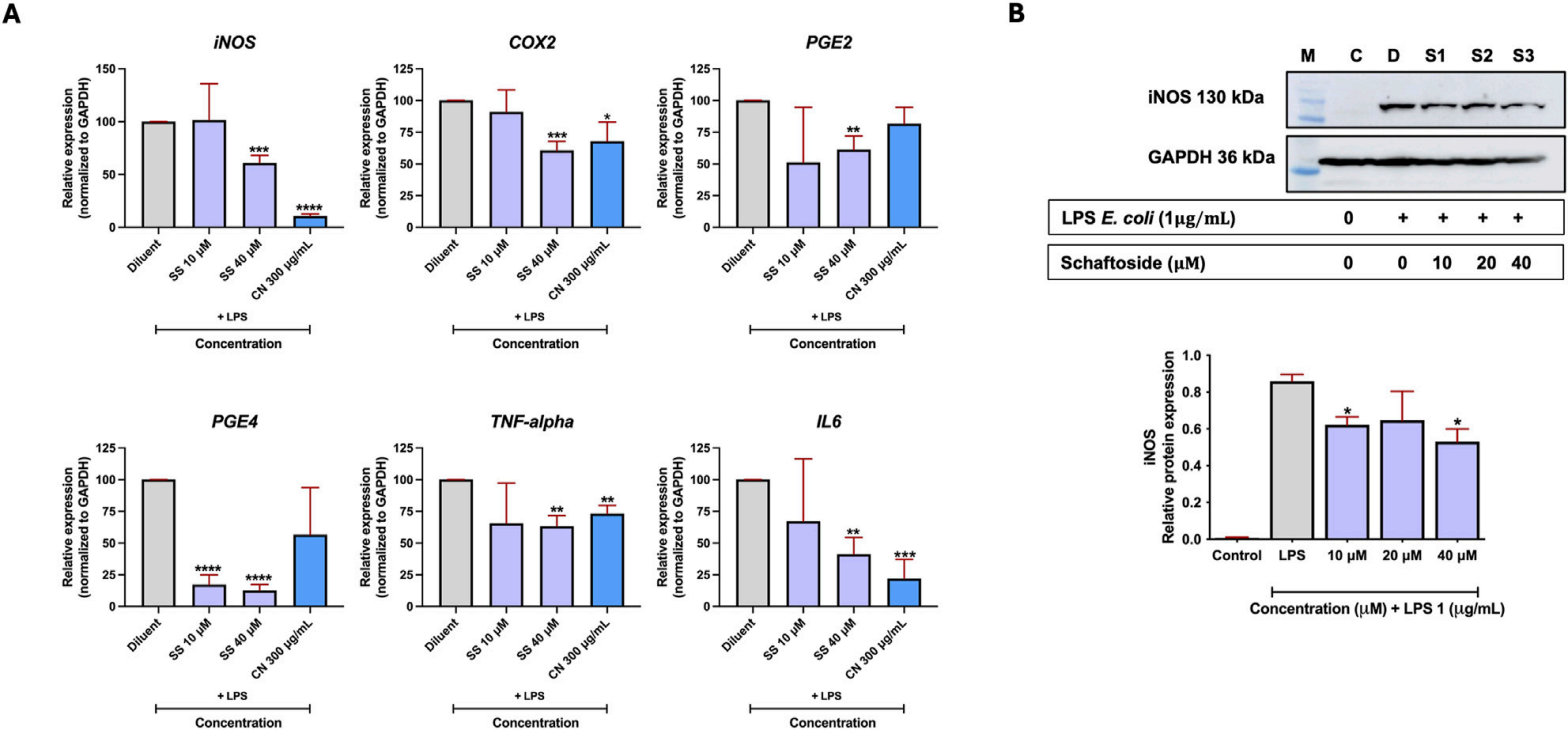
The effect of schaftoside on reducing mRNA and protein expression levels of inflammatory markers in LPS-induced RAW 264.7 cells. **(A)** The mRNA expression levels of genes related to inflammation (*iNOS, COX2, PGE2, PGE4, TNF-α*, and *IL6*) in RAW 264.7 cells following LPS-induced inflammation and treatment with schaftoside or *C. nutans* extract (CM03 -300 μg/mL, used as a positive control). **(B)** The iNOS protein expression level in RAW 264.7 cells following LPS-induced inflammation and treatment with schaftoside, normalized to GAPDH (housekeeping protein). Relative expression was calculated with the LPS treatment group (without schaftoside or *C. nutans*) set as 100%. Asterisk indicates significant difference: (ns) indicates *p* > 0.05, *p < 0.05, **p < 0.01, ***p < 0.001, ****p < 0.0001.

To investigate the effect of schaftoside in reducing LPS-induced inflammation, real-time PCR was used to quantify the expression of key genes related to inflammation in RAW 264.7 cells. The cells were treated with 10 and 40 μM schaftoside after the LPS induction. The results demonstrated that schaftoside at 40 μM significantly reduced the expression of all tested genes: *iNOS, COX2, PGE2, PGE4, TNF-α*, and *IL6*. However, at a lower concentration (10 μM), only PGE4 showed a significant reduction in its expression. Interestingly, *C. nutans* extract (CM03) at 300 μg/mL, used as a positive control, significantly reduced the expression levels of *iNOS, COX2, TNF-α,* and *IL6,* with the most pronounced reduction observed in *iNOS* expression (Figure 2A).

Therefore, immunoblotting for iNOS was performed to evaluate its protein expression levels. Similarly, inflammation in RAW 264.7 cells was induced using *E. coli* LPS, and the cells were subsequently treated with different concentrations of schaftoside (10, 20, and 40 μM). The results showed a decrease in iNOS protein in schaftoside-treated cells, with significant reductions observed at 10 and 40 μM and, though not significant, the reduction was also observed at 20 μM (Figure 2B; Supplemetary Figure S1). It should also be noted that schaftoside at concentrations of 6.25–200 μM on RAW 264.7 cells did not exhibit obvious cytotoxicity (Supplementary Figure S2).

### 3.2 *C. nutans* identification and their cytotoxicity

**FIGURE 3:**
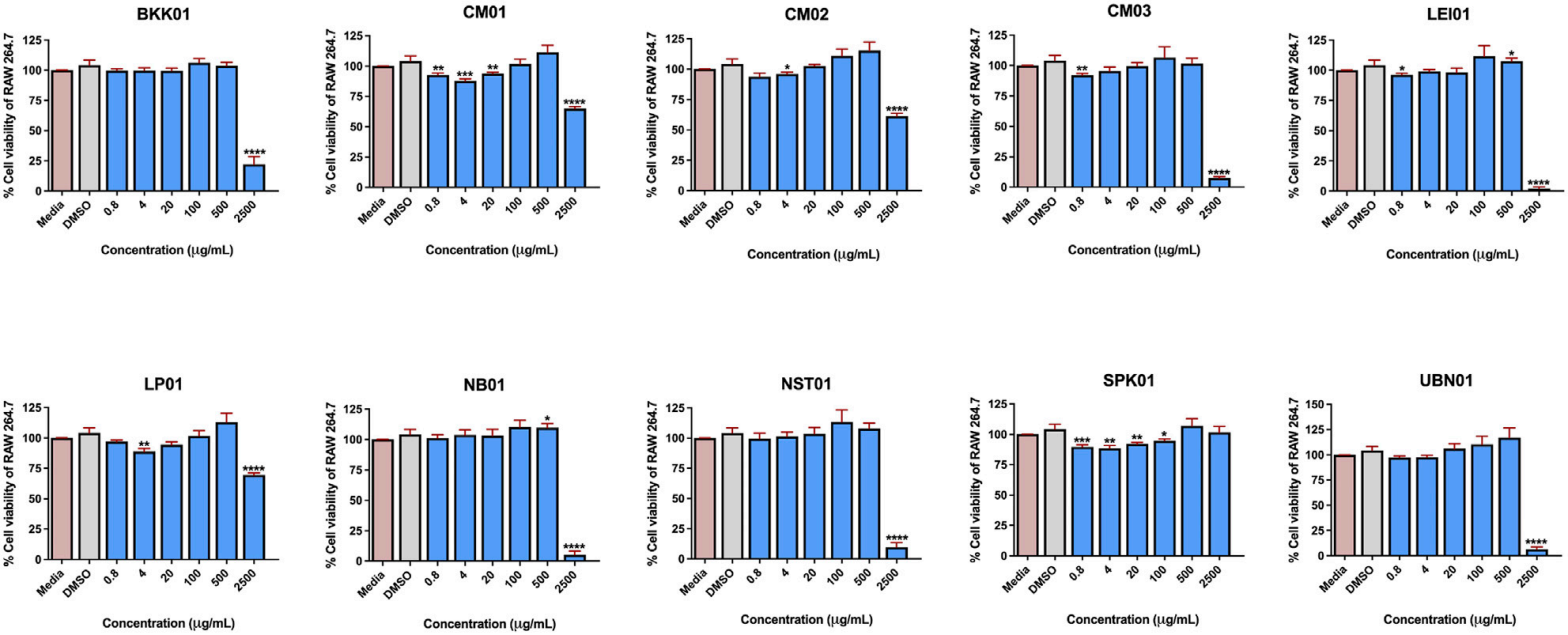
The effect of *C. nutans* extracts on RAW 264.7 cell lines. The cells were treated with *C. nutans* extracts at a concentration of 0.8–2,500 μg/mL (2-fold dilution) and was monitored after 24 h by using PrestoBLUE™ cell viability reagent. The percentage of cell viability was analyzed relative to the non-treatment control set as a 100%. Indicates significant difference: (ns) indicates *p* < 0.1234, *p < 0.0332, **p < 0.0021, ***p < 0.0002, and ****p < 0.0001.

Our previous work demonstrated that genetic diversity of *C. nutans* resulted in different bioactivity, particularly on anti-apoptotic, antioxidant, and anti-bacterial activities (Chiangchin et al., 2023). In this work, *C. nutans* deposited to the Queen Sirikit Botanic Garden Herbarium (QSBG herbarium) from ten different locations in Thailand were further assessed. The detailed information is presented in Supplementary Table S2. The dried leaves of all plant samples were extracted using 95% ethanol. The cytotoxicity of all *C. nutans* extracts at the concentration varied from 0.8-2,500 μg/mL on RAW 264.7 cells were evaluated at 24 h. The results suggested that the extract from each plant exhibited no cytotoxicity at a concentration of 500 μg/mL relative to that of the non-treated control after 24 h (Figure 3).

### 3.3 Effect of *C. nutans* ethanolic extracts on iNOS expression

**FIGURE 4:**
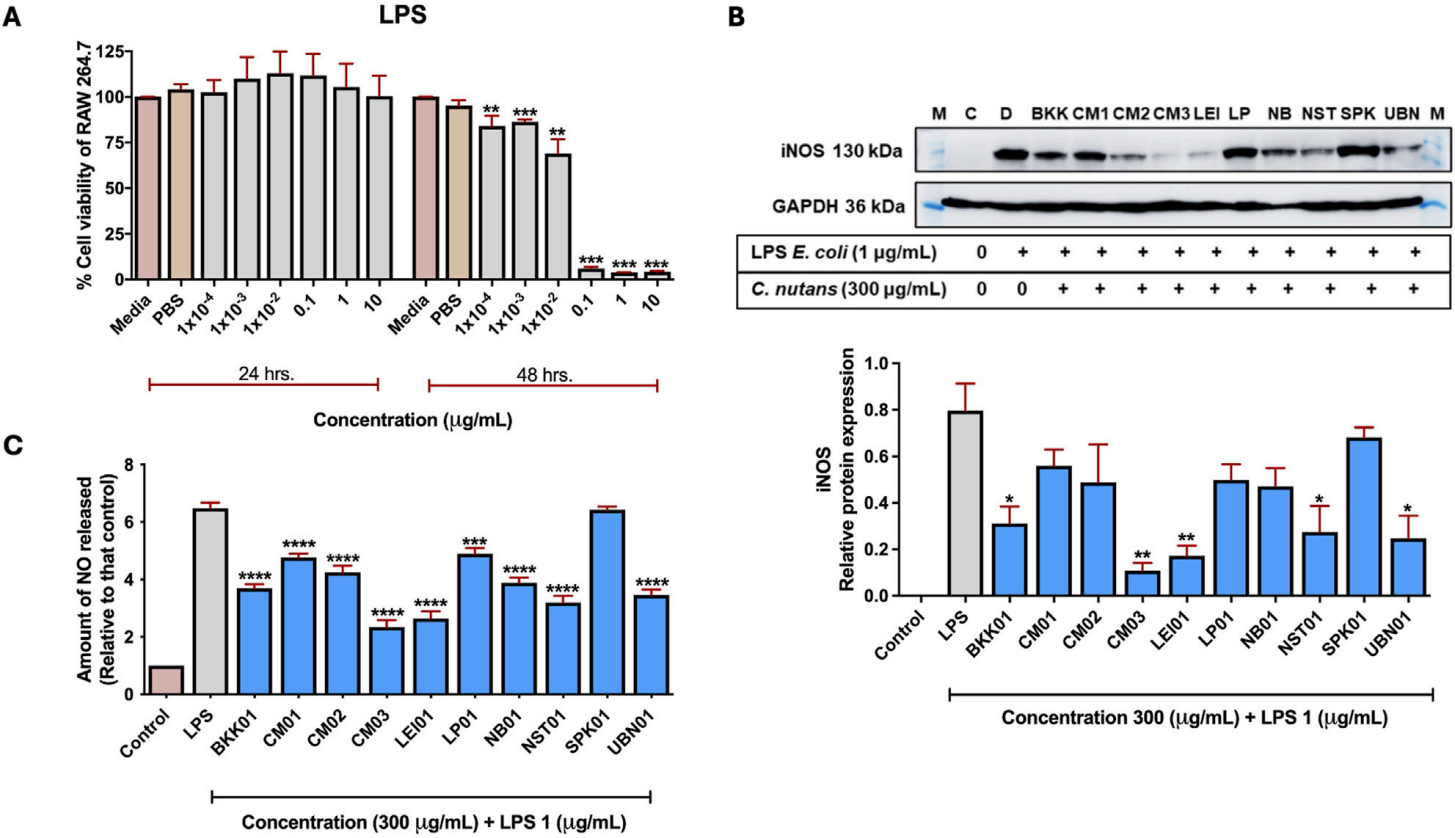
The effect of *C. nutans* extracts on reducing inflammation on iNOS protein expression and NO release in LPS-induced RAW 264.7 cells. **(A)** The cytotoxicity of LPS at concentrations of 1 × 10^-4^–10 μg/mL on RAW 264.7 cells for 24 and 48 h using Prestoblue™ cell viability reagent. **(B)** Expression of iNOS protein with the highest expression LPS-induced (1 μg/mL) and the effect of *C. nutans* extracts (300 μg/mL) on inhibiting the high-level expression of iNOS protein validated using Western blot analysis. The fold change of iNOS protein expression was normalized with GAPDH (housekeeping protein) and calculated as the relative expression to LPS treatment without *C. nutans* extracts. **(C)** The effect of *C. nutans* extracts (300 μg/mL) on reducing NO release after LPS-induction for 24 h validated using Gress reagent. Arterisk indicates significant difference: (ns) indicates *p* > 0.05, *p < 0.05, **p < 0.01, ***p < 0.001, ****p < 0.0001.

Since iNOS is a key protein involved in the inflammatory response, its elevation indicates the presence of inflammation and as shown previously that iNOS showed the most pronounced effect as a result of schaftoside treatment, the effect of *C. nutans* extracts from different locations on reducing LPS-induced iNOS protein expression in RAW 264.7 cells were investigated. Initially, we determine the optimal dose of lipopolysaccharide (LPS) that could induce an inflammatory response in RAW 264.7 cells without compromising cell viability. A cytotoxicity assay was performed by treating RAW 264.7 cells with various concentrations of LPS for 24 and 48 h. The results suggested that at different concentrations (1 × 10^-4^–10 μg/mL), LPS did not show cytotoxicity to the cells after 24 h of treatment, however, at the same range of concentrations, LPS significantly reduce the cell viability at 48 h (Figure 4A). Therefore, the 24 h incubation and 1 μg/mL of LPS were further used in following studies. The iNOS expression was used to screen for the most promising *C. nutans* extracts with anti- inflammatory activity. Additionally, the reduction of nitric oxide (NO) release from the cells was assessed in parallel with iNOS expression levels. iNOS expression significantly decreased following treatment with 300 μg/mL *C. nutans* extracts from BKK01, CM03, LEI01, NST01, and UBN01. While the remaining extracts did not exhibit significant reductions, a decreasing trend in iNOS expression was observed across all samples, suggesting an overall reduction in iNOS levels (Figure 4B; Supplementary Figure S3). Furthermore, all *C. nutans* extracts demonstrated a corresponding reduction in NO release, confirming decreased iNOS activity (Figure 4C). However, treatments of *C. nutans* extract with COX2 expression did not show significantly decreased expression (Supplementary Figure S4).

### 3.4 Quantification of schaftoside in *C. nutans* extracts from different locations

**FIGURE 5:**
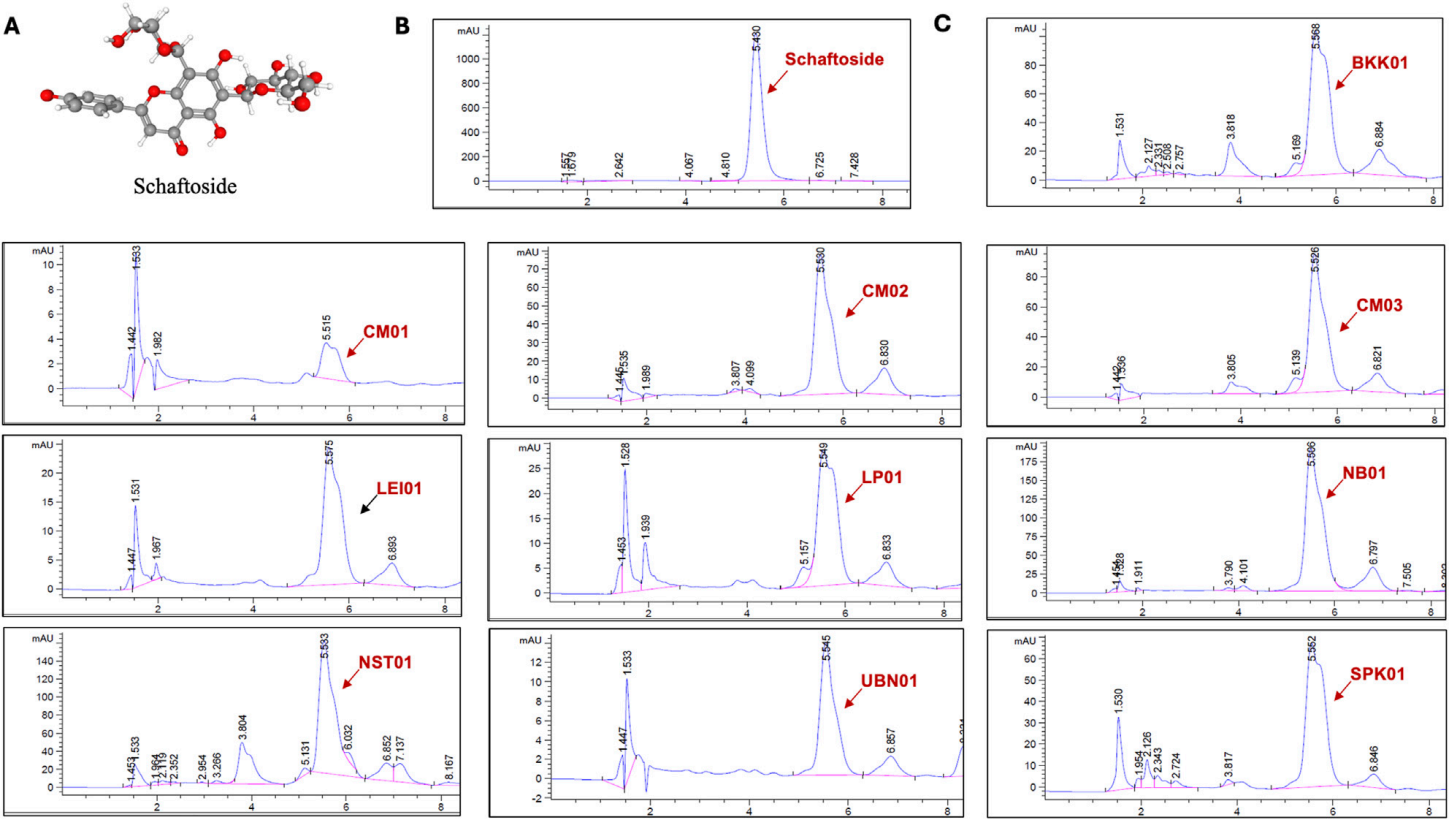
The chromatograms of *C. nutans* extracts at absorbance 340 nm using HPLC analysis. **(A)** Chemical structure of schaftoside **(B)** HPLC chromatograms of schaftoside standard **(C)** HPLC chromatograms of *C. nutans* extracts from different locations.

Subsequently, the amount of schaftoside in all extracts was quantified using HPLC (Figure 5) and showed that the *C. nutans* extract NB01 contained the highest amount of schaftoside, following by NST01 and BKK01, respectively (Table 1). Interestingly, this did not corelate with the anti-inflammatory activity as NB01 did not show the significant activity in reducing iNOS expression level shown previously suggesting that the observed activity may be the combined effects from different bioactive compounds present in the extracts.

**TABLE 1:** Amount of schaftoside in *C. nutans* extracts using HPLC analysis.

*C. nutans* extracts samples	Amount of schaftoside (mg schaftoside/g extracts)
1. *Clinacanthus nutans* (BKK01)	13.186 ± 0.248
2. *Clinacanthus nutans* (CM01)	0.055 ± 0.003
3. *Clinacanthus nutans* (CM02)	8.194 ± 0.048
4. *Clinacanthus nutans* (CM03)	9.955 ± 0.199
5. *Clinacanthus nutans* (LEI01)	2.715 ± 0.013
6. *Clinacanthus nutans* (LP01)	3.283 ± 0.025
7. *Clinacanthus nutans* (NB01)	22.250 ± 0.159
8. *Clinacanthus nutans* (NST01)	16.855 ± 0.613
9. *Clinacanthus nutans* (SPK01)	8.539 ± 0.051
10. *Clinacanthus nutans* (UNB01)	1.135 ± 0.011

As it was suggested that schaftoside may not be the only bioactive compounds in *C. nutans* extracts, the total phenolic content of each extract was then evaluated. Interestingly, NST01 showed the highest total phenolic content with 14.002 mg GAE/g extracts following by LEI01 with 11.949 mg GAE/g extract and CM03 with 11.857 mg GAE/g extract, respectively (Table 2). This further suggested that other phenolic compounds may play synergistic roles in reducing the inflammation rather than schaftoside alone.

**TABLE 2:** The total phenolic content in *C. nutans* leaf extracts. Each value of the total phenolic content was calculated with a standard curve of gallic acid.

*C. nutans* extracts samples	Amount of total phenolic content (mg GAE/g extracts)
1. *Clinacanthus nutans* (BKK01)	8.905
2. *Clinacanthus nutans* (CM01)	8.650
3. *Clinacanthus nutans* (CM02)	10.310
4. *Clinacanthus nutans* (CM03)	11.857
5. *Clinacanthus nutans* (LEI01)	11.949
6. *Clinacanthus nutans* (LP01)	7.820
7. *Clinacanthus nutans* (NB01)	9.192
8. *Clinacanthus nutans* (NST01)	14.002
9. *Clinacanthus nutans* (SPK01)	6.716
10. *Clinacanthus nutans* (UNB01)	9.182

### 3.5 Molecular docking

**FIGURE 6:**
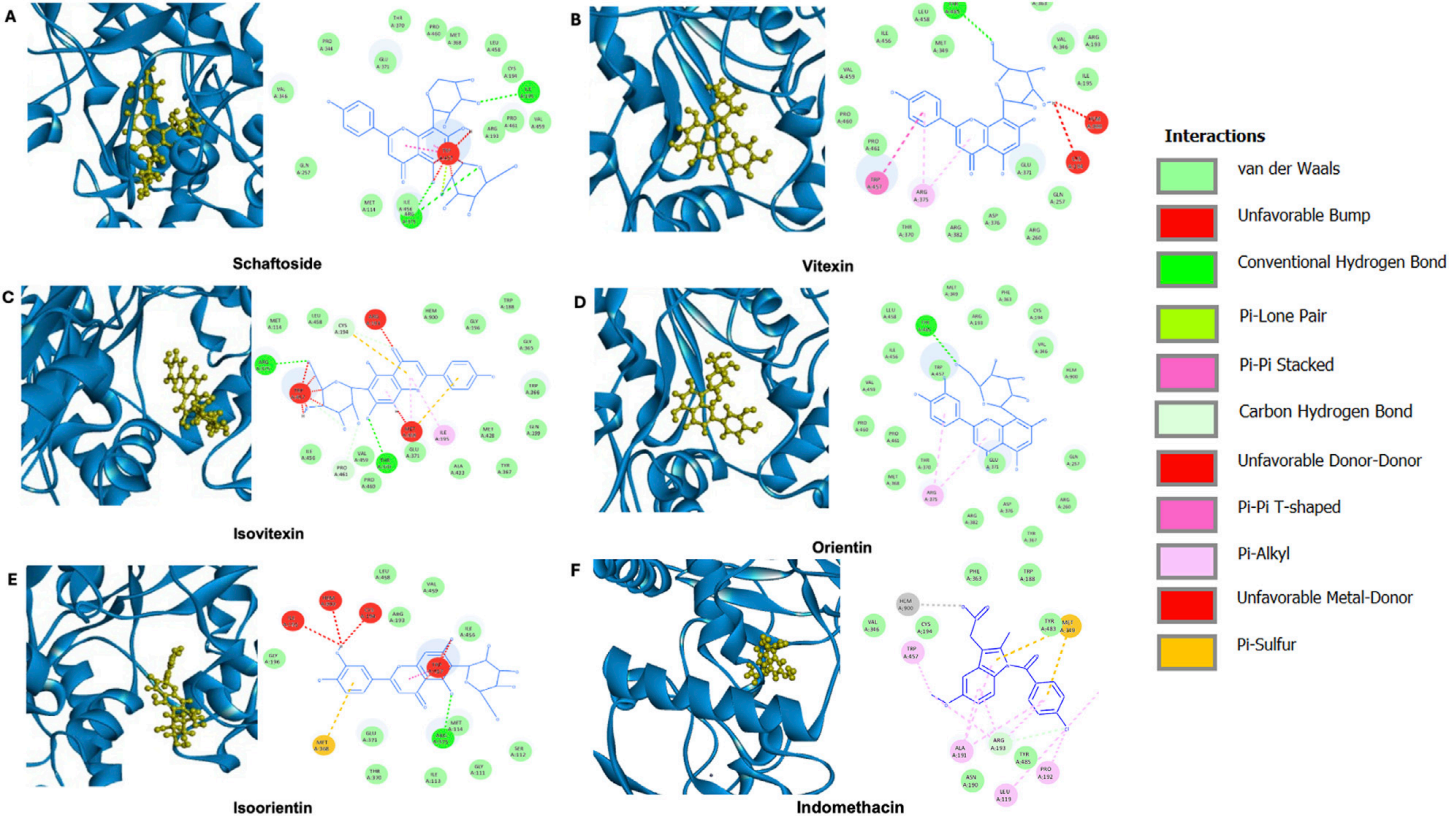
Molecular docking analysis of potential bioactive compounds in *C. nutans* with iNOS protein. Docking poses of iNOS protein (PDB ID IQW4) with **(A)** schaftoside in comparison with the potential bioactive compounds from *C. nutans* extracts including **(B)** vitexin, **(C)** isoorientin, **(D)** orientin, **(E)** isovitexin, and **(F)** indomethacin.

Other compounds present in *C. nutans* have been reported for their anti-inflammatory effects in addition to schaftoside. Notably, vitexin, isoorientin, orientin, and isovitexin have been documented for their potential roles (Chelyn et al., 2014). In this study, molecular docking analysis was performed to evaluate the binding of these compounds to iNOS, a key protein involved in inflammatory processes and the protein that showed the most pronounced effects upon *C. nutans* extract treatment (Figures 6A-F). The results indicated that the fitness scores (Table 3) for isoorientin and isovitexin were higher than that of schaftoside, suggesting that their binding interactions with iNOS may significantly contribute to the observed anti-inflammatory effects. Indomethacin (standard anti-inflammatory drug) were highest the fitness scores than that five bioactive compounds in *C. nutans*. Schaftoside has shown to interact with iNOS through conventional hydrogen bonds at ILE195, ARG375, and ILE456 (Figure 6A). In comparison, isovitexin, which achieved the highest fitness score, formed conventional hydrogen bonds at THR370 and ARG375 (Figure 6C). Isoorientin demonstrated interactions with iNOS via a conventional hydrogen bond at ARG375 and a Pi-sulfur bond at MET368 (Figure 6E). Interestingly, self-docking with the co-crystallized ligand (protoporphyrin IX containing Fe) showed a lower fitness score, further supporting the hypothesis that these compounds may bind effectively to iNOS and influence its activity (Table 3).

**TABLE 3:** Fitness score from molecular docking of iNOS protein with different potential bioactive compounds.

Bioactive compound	The fitness of the top-ranked (best ranking) Ligand = 3 A R; N-OMEGA-PROPYL-L-ARGININE (10°A)
1. Schaftoside	61.77
2. Vetixin	59.14
3. Isovetixin	63.45
4. Orientin	61.28
5. Isoorientin	63.10
6. Indomethacin	79.98
7. Self-docking	47.50

## 4 Discussion

In Thai folk medicine, the fresh leaves of *C. nutans*, a plant listed in the Thai Herbal Pharmacopoeias, are traditionally used to treat skin injuries, including snake bites and insect bites (Alam et al., 2016). This traditional use highlights its potential anti-inflammatory properties, as these conditions often involve localized inflammation. However, a thorough investigation on the mechanisms of bioactive compounds in *C. nutans* have been limited. Schaftoside is one of the major flavone compounds with a handful number of reports demonstrating its anti-inflammatory effects. In this work, several key genes related to inflammation signaling pathways were used to investigate the effects of schaftoside on inflammation reduction in RAW 264.7 murine macrophage. Our results clearly demonstrated that schaftoside treatment at 40 μM effectively reduced the expression of all key genes related to inflammation signaling pathways *iNOS, COX2, PGE2, PGE4, TNF-α,* and *IL6* in LPS-treated RAW 264.7 cells. This indicates that schaftoside suppresses inflammatory responses at the mRNA level. Among these, iNOS exhibited the most significant reduction, leading us to further investigate its expression at the protein level using immunoblotting assay. The results confirmed a decrease in iNOS protein expression, suggesting that schaftoside mitigates inflammation through the NF-κB signaling pathway (Adel et al., 2024).

The variation in the biological activity of *Clinacanthus nutans* across different geographic regions is primarily influenced by the interplay between genetic diversity and environmental factors. Genetic differentiation among populations can result in variability in the biosynthesis of secondary metabolites, which are key contributors to pharmacological effects such as antioxidant, anti-inflammatory, and antibacterial activities. Moreover, environmental factors including soil composition, climate, altitude, and local microbial interactions can modulate gene expression involved in metabolite production (Chiangchin et al., 2023; Elansary et al., 2020). These combined influences lead to significant differences in the phytochemical profiles and bioactivities of *C. nutans* collected from various regions, highlighting the importance of geographic origin in medicinal plant research and application. In our study, ten ethanolic extracts of *C. nutans* collected from different regions of Thailand demonstrated the ability to mitigate LPS-induced inflammation in RAW 264.7 cells. However, despite being tested at the same concentration, the degree of anti-inflammatory efficacy varied among the extracts, suggesting differences in their phytochemical composition. Notably, our previous genetic analysis revealed that *C. nutans* from different geographic regions of Thailand share closely related genetic backgrounds (Chiangchin et al., 2023), further supporting the hypothesis that environmental factors may play a predominant role in shaping the chemical profiles and resulting bioactivity of *C. nutans* extracts.

The chemical profiles of medicinal plants have been successfully utilized as biomarkers to assess their bioactivities as therapeutic agents. Groups chemicals are employed as quality biomarkers, necessitating the identification and quantification of multiple compounds (Osman et al., 2019). However, the use of a single compound as a biomarker has also been proposed for practicality and precision. For instance, artemisinin from *Herba Artemisiae* Annuae (Qinghao) serves as a chemical biomarker for anti-malaria activity, detectable through various analytical techniques (Li et al., 2008). Among those bioactive compounds of *C. nutans* extract, schaftoside was the focus due to its report as a major flavone present in *C. nutans* ethanolic extracts (Chelyn et al., 2014). We thus investigated the correlation between the schaftoside and anti-inflammatory activities of *C. nutans* ethanolic extracts collected from 10 different regions. Unfortunately, in our study the amount of schaftoside in different extracts did not corelate with their anti-inflammatory activity. While CM03 exhibited the lowest iNOS expression, its schaftoside content was measured at 9.9 mg/g extract. In contrast, NB01, which contained the highest amount of schaftoside (22.2 mg/g extract), did not show a significant reduction in iNOS expression. This discrepancy suggests that factors beyond schaftoside concentration, such as the presence of other bioactive compounds or synergistic interactions, may play a critical role in the anti-inflammatory activity of *C. nutans* extracts. A recent work has detailed synergistic anti-inflammatory effects by combining more than one phytochemicals as results of regulating multiple pathways, multiple cells, and inflammatory markers (Zhang et al., 2019). The total phenolic content in all *C. nutans* samples was subsequently determined, revealing that NST01 contained the highest phenolic content, which corresponded to its ability to reduce iNOS expression. CM03, while exhibiting significant iNOS reduction, showed a phenolic content of 11.8 mg GAE/g extract. These findings suggest that bioactive compounds other than schaftoside such as isovitexin, orientin, isoorientin, and vitexin may contribute to the biological activities of *C*. *nutans*. These flavonoids are believed to exert their effects through suppression of the NF-κB signaling pathway and antioxidant activity, thereby supporting the plant’s traditional use in managing inflammation-related conditions (Ng et al., 2020). This study also confirmed that the geographic origin of the plant influences its bioactive compound profile, particularly schaftoside content and total phenolic content (TPC). This observation aligns with the findings of Chiangchin et al. (2023), who demonstrated that variation in the biological activity of *C. nutans* across different regions is driven by a complex interplay of genetic diversity and environmental factors, which collectively modulate the synthesis of bioactive compounds. Therefore, relying on schaftoside alone as a chemical marker for quality control may be inadequate for *C. nutans*.

A previous study performed molecular docking of iNOS with benzimidazole-coumarin derivatives as potent inhibitors with selective iNOS targeting and significant anti-inflammatory activity (Minhas and Bansal, 2022). This suggests that the importance to investigating the binding between compounds with iNOS protein. Therefore, further investigations of the bioactive compounds were conducted using molecular docking comparing between schaftoside and other potential bioactive candidates in *C. nutans* ethanolic extracts. Molecular docking of iNOS protein with isoorientin and isovitexin indicated a high likelihood of protein-compound binding. A previous study (Lv et al., 2016) suggested that isovitexin can reduce pro-inflammatory cytokine secretion, iNOS and COX-2 expression, and decrease ROS generation *in vitro*. While the anti-inflammatory properties of isoorientin have been reported (Anilkumar et al., 2017; Li et al., 2020), however, to the best of our knowledge, none of the reports has demonstrated the binding of these compounds and iNOS protein. This study comprehensively identified key inflammation-related genes induced by LPS that were mitigated by schaftoside. The relationship between schaftoside content and iNOS expression reduction across different *C. nutans* extracts was also explored, suggesting that schaftoside is not the sole bioactive compound contributing to the plant’s anti-inflammatory properties. Furthermore, *in silico* analysis revealed that isoorientin and isovitexin exhibited stronger binding to the iNOS protein, highlighting their potential as iNOS inhibitors. However, when compared to the established anti-inflammatory drug indomethacin, all five bioactive compounds identified in *C. nutans* demonstrated lower binding efficiency. These findings pave the way for further in-depth investigations of these compounds, both individually and synergistically with schaftoside.

## Data Availability

The original contributions presented in the study are included in the article/Supplementary Material, further inquiries can be directed to the corresponding author.
