# Supplementary material for: Schaftoside contributed to anti-inflammatory activity of Clinacanthus nutans extract in lipopolysaccharide-induced RAW 264.7 cells
Source: Front Pharmacol. 2025 Jun 10;16:1584620. doi: 10.3389/fphar.2025.1584620 (PMC12186155; doi:10.3389/fphar.2025.1584620)
Supplement: Supplementary file 1 [file DataSheet1.docx]

**Supplementary information**

**Supplementary Figure S1.** Full blots were derived from immunoblotting assay. The square frame indicates cropped area of interested proteins that were exhibited in figure 2B.

(C = Control, D = LPS + DMSO, S1 = LPS + Schaftoside 10 μM, S2 = LPS + Schaftoside 20 μM, and S3 = LPS + Schaftoside 40 μM)


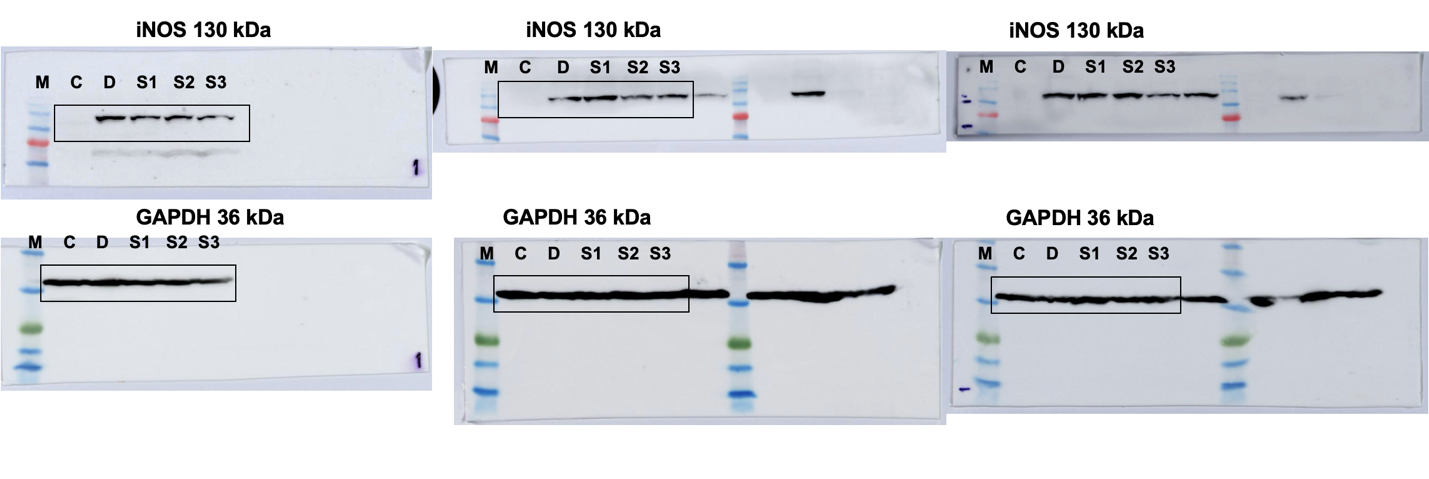


**Supplementary Figure S2.** The cytotoxicity of schaftoside at a concentration of 6.25 - 200 μM on RAW 264.7 cell lines for 24 h using Prestoblue^TM^ cell viability reagent.


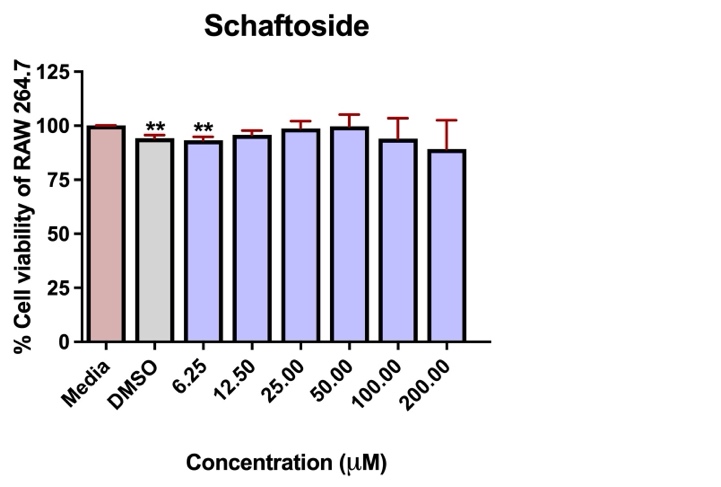


**Supplementary Figure S3.** Full blots were derived from immunoblotting assay. The square frame indicates cropped area of interested proteins that were exhibited in figure 3B.

(C = Control, D = LPS + DMSO, 10 sample of *C. nutans* 300 μg/mL + LPS)


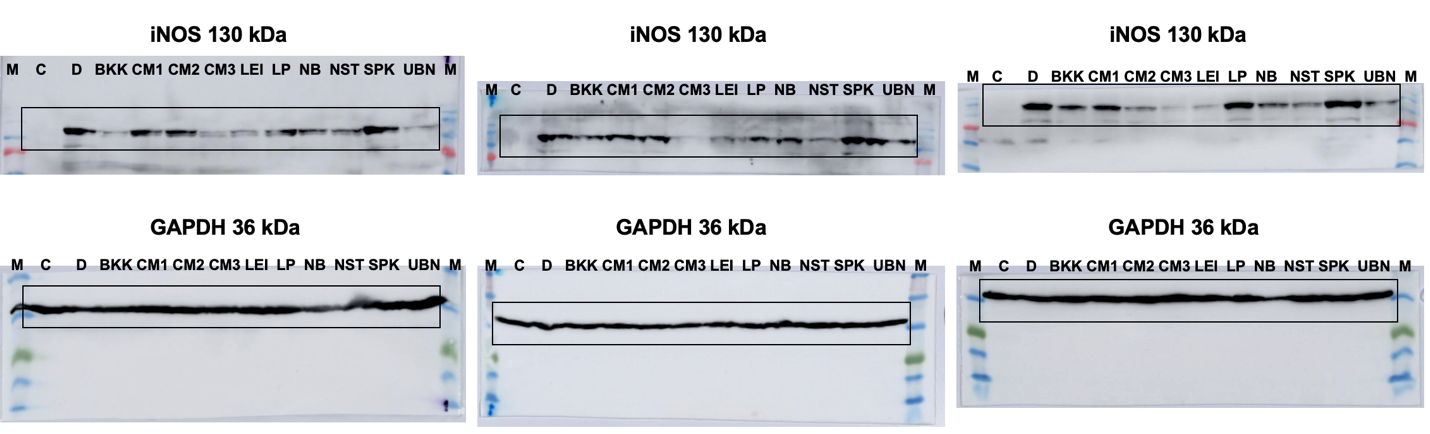


**Supplementary Figure S4.** The effect of *C. nutans* extracts and schaftoside on reducing inflammation in COX2 protein expression levels when LPS-induced RAW 264.7 cell lines.


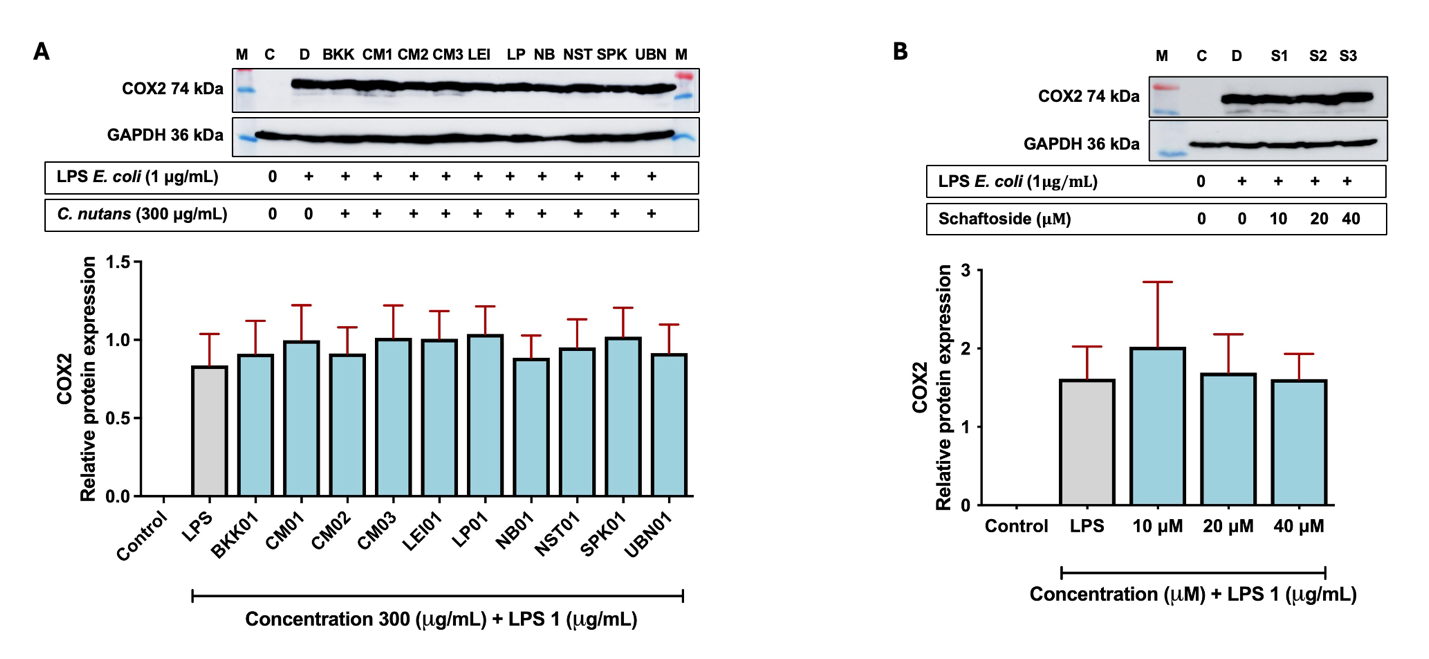


**Supplementary Table S1.** List of real-time PCR primers. (Kim et al., 2013; González et al., 2022; Zhang et al., 2018; Cifuentes et al., 2018)

| **Genes** | **Forward primer (5'→3')** | **Reverse primer (5'→3')** |
| --- | --- | --- |
| *iNOS* | 5′ CATGATGGTCACATTCTGC 3′ | 5′ GTCTTGCAAGCTGATGGTC 3′ |
| *COX2* | 5′ GAGTCCATGTTCCAGGAGGA 3′ | 5′ CCCCACAGTCAAAGACACT 3′ |
| *PGE2* | 5′ GGCAAGGAGCATATGGCGAAGGTG 3′ | 5′ GTGGCCCTGGCTCCCGAAAGTC 3′ |
| *PGE4* | 5′ AACACTTTTGGCCTGAACTTGT 3′ | 5′ AGTAGCTAAAGGGGGAATCTT 3′ |
| *TNF-alpha* | 5′ CGGACTCCGCAAAGTCTAAG 3′ | 5′ CGTCAGCCGATTTGCTATCT3′ |
| *IL6* | 5′ GGAAATTGGGGTAGGAAGGA 3′ | 5′ CCGGAGAGGAGACTTCACAG 3′ |
| *GADPH* | 5′ GTCAGATCCACGACGGACACATT 3′ | 5′ CAGGAGCGAGACCCCACTAACAT 3′ |

**Supplementary Table S2.** Geographical locations of *C. nutans* in eight provinces in Thailand and voucher numbers.

| **Plant samples (code ID)** | **Geographical location** | **Voucher numbers** |
| --- | --- | --- |
| 1. *Clinacanthus nutans* (BKK01) | Bangkok province, Thailand | WP9177 |
| 2. *Clinacanthus nutans* (CM01) | Chiang Mai province, Thailand | WP9178 |
| 3. *Clinacanthus nutans* (CM02) | Chiang Mai province, Thailand | WP9179 |
| 4. *Clinacanthus nutans* (CM03) | Chiang Mai province, Thailand | WP9180 |
| 5. *Clinacanthus nutans* (LEI01) | Lei province, Thailand | WP9181 |
| 6. *Clinacanthus nutans* (LP01) | Lampang province, Thailand | WP9182 |
| 7. *Clinacanthus nutans* (NB01) | Nonthaburi province, Thailand | WP9183 |
| 8. *Clinacanthus nutans* (NST01) | Nakhon Si Thammarat province, Thailand | WP9184 |
| 9. *Clinacanthus nutans* (SPK01) | Samut Prakan province, Thailand | WP9185 |
| 10. *Clinacanthus nutans* (UNB01) | Ubon Ratchathani province, Thailand | WP9186 |

**References**

Cifuentes, Adriana, Claudia González, María Isabel Rodríguez, and Andrés Muñoz. 2018. “In Vitro Anti-Inflammatory Activity of Plant Extracts in LPS-Stimulated RAW 264.7 Macrophages.” Journal of Ethnopharmacology 214:123–131. doi: 10.1016/j.jep.2017.12.043.

González-Montoya, Mónica, Beatriz Hernández-Ledesma, José M. Silván, and Rosa Mora-Escobedo. 2022. “Anti-Inflammatory Activity of Protein Hydrolysates from Common Beans on LPS-Stimulated RAW 264.7 Macrophages.” Food & Function 13(3):1582–1591. doi: 10.1039/D1FO03745K.

Kim, Young-Mi, Tae-Hyun Kim, and Yong-Chul Chung. 2013. “Modulation of Inflammatory Responses by Curcumin in RAW 264.7 Cells: Involvement of the NF-κB Signaling Pathway.” International Immunopharmacology 17(2):385–392. doi: 10.1016/j.intimp.2013.07.011.

Zhang, Yan, Dongmei Liu, and Lili Wang. 2018. “Anti-Inflammatory Effects of Natural Compounds in LPS-Stimulated RAW 264.7 Macrophages.” Mediators of Inflammation 2018: Article ID 2454839. doi: 10.1155/2018/2454839.
